# Hepatic Carboxylesterase 1 Is Induced by Glucose and Regulates Postprandial Glucose Levels

**DOI:** 10.1371/journal.pone.0109663

**Published:** 2014-10-06

**Authors:** Jiesi Xu, Liya Yin, Yang Xu, Yuanyuan Li, Munaf Zalzala, Gang Cheng, Yanqiao Zhang

**Affiliations:** 1 Department of Integrative Medical Sciences, Northeast Ohio Medical University, Rootstown, Ohio, United States of America; 2 Department of Pharmacology and Toxicology, College of Pharmacy, University of Baghdad, Baghdad, Iraq; 3 Department of Chemical and Biomolecular Engineering, University of Akron, Akron, Ohio, United States of America; University of Toronto, Canada

## Abstract

Metabolic syndrome, characterized by obesity, hyperglycemia, dyslipidemia and hypertension, increases the risks for cardiovascular disease, diabetes and stroke. Carboxylesterase 1 (CES1) is an enzyme that hydrolyzes triglycerides and cholesterol esters, and is important for lipid metabolism. Our previous data show that over-expression of mouse hepatic CES1 lowers plasma glucose levels and improves insulin sensitivity in diabetic o*b*/*ob* mice. In the present study, we determined the physiological role of hepatic CES1 in glucose homeostasis. Hepatic CES1 expression was reduced by fasting but increased in diabetic mice. Treatment of mice with glucose induced hepatic CES1 expression. Consistent with the in vivo study, glucose stimulated CES1 promoter activity and increased acetylation of histone 3 and histone 4 in the CES1 chromatin. Knockdown of ATP-citrate lyase (ACL), an enzyme that regulates histone acetylation, abolished glucose-mediated histone acetylation in the CES1 chromatin and glucose-induced hepatic CES1 expression. Finally, knockdown of hepatic CES1 significantly increased postprandial blood glucose levels. In conclusion, the present study uncovers a novel glucose-CES1-glucose pathway which may play an important role in regulating postprandial blood glucose levels.

## Introduction

Metabolic syndrome refers to a group of metabolic disturbances, including obesity, hyperglycemia, dyslipidemia and hypertension, which increase the risks for cardiovascular disease, diabetes and stroke. The prevalence of metabolic syndrome is estimated to be 34% of the U.S. adult population and the prevalence has risen since people adopt the unhealthy dietary and inactive lifestyle. Lipid and glucose metabolism is tightly regulated in the body by modulating their dietary intake, transport, synthesis, storage and elimination. Any disturbances of these metabolic processes may increase the risks for metabolic diseases.

Carboxylesterase 1 (CES1) is a drug-metabolizing enzyme that is highly expressed in the liver but also to a lesser extent in the intestine, macrophages and other tissues. CES1 catalyzes the hydrolytic reaction with the release of alcohol substituent and acyl group-containing molecule from the substrate [1]. CES1 possesses triglyceride (TG) and cholesterol ester (CE) hydrolase activity [2], [3], [4], [5], and is shown to play an important role in regulating lipid metabolism [4], [5], [6], [7], [8]. *Ces1* ^−/−^ mice developed obesity, fatty liver, hyperinsulinemia, and insulin insensitivity, thus highlighting the importance of CES1 in lipid metabolism [6]. CES1 has been shown to prevent TG accumulation in rat McArdle-RH7777 hepatocytes [4]. The intestinal CES1 regulates chylomicron assembly, secretion and clearance [9]. Our recent data show that adenovirus-mediated over-expression of mouse CES1 (Ad-CES1) lowers hepatic TG and plasma glucose levels in both wild-type and diabetic mice and improves glucose tolerance in diabetic mice [5]. These latter data indicate that CES1 plays an important role in glucose metabolism.

Glucose is a major energy source for the body to cope with nutrient deprivation [10]. Blood glucose level is tightly controlled to maintain systemic glucose homeostasis [10]. When blood glucose level rises, a number of events, including glucose transport, glycolysis, glycogenesis, lipogenesis and gluconeogenesis, are coordinately regulated to keep blood glucose level within a normal range [11], [12]. Glucose has been shown to directly regulate the transcription of genes involved in the conversion of carbohydrates to lipids in the liver [13], [14]. Carbohydrate response element-binding protein (ChREBP) mediates glucose-induced gene expression in the liver [15], [16], [17]. In response to glucose stimulation, ChREBP is activated and binds to carbohydrate response element (ChoRE) in the promoter of liver-type pyruvate kinase (*L-PK*), a glycolytic gene, and lipogenic genes, including acetyl-CoA carboxylase (*ACC*) and fatty acid synthase (*FAS*). In contrast, ChREBP is inactivated under starvation conditions, suggesting that ChREBP can sense blood glucose levels [18]. Liver X receptor (LXR) is reported to be another glucose sensor, which integrates hepatic glucose metabolism and fatty acid synthesis [19]. Recent data have shown that glucose induction of gene transcription is associated with epigenetic modifications of histone [20], [21]. ATP-citrate lyase (ACL) converts glucose-derived citrate to acetyl-CoA, a substrate for histone acetyltransferases which acetylate the lysine residue (K) in the histone 3 (H3) and H4 tails [21]. Therefore, nuclear ACL is required for glucose-mediated histone acetylation and gene activation [21], [22].

Modulation of postprandial glucose levels plays an important role in glycemic control. In humans, postprandial glucose levels peak at ∼ 1 h after the start of the meal and then return to preprandial levels within 2–3 h [23], [24]. A link between high postprandial glycemic levels and the development of cardiovascular diseases (CVD) and diabetes underscores the significance of controlling postprandial glucose [25]. Our previous data [5] and data from Quiroga *et al*. [6] suggest that CES1 may play a role in the control of blood glucose levels. Whether CES1 regulates postprandial glucose levels remains unknown. In the present study, we determined the reciprocal regulation between glucose and the expression of mouse CES1 (also called Ces1g, Es-x) as well as the role of hepatic CES1 in postprandial glucose control. Our data suggest a novel glucose-CES1-glucose pathway that may play an important role in regulating postprandial glucose levels.

## Material and Methods

### Mice, diets and gavage

C57BL/6 mice, *ob/ob* mice and *db/db* mice were purchased from the Jackson Laboratories (Bar Harbor, ME). High fat/high cholesterol (HFHC) diet (21% kcal from fat, 1.5% cholesterol) was purchased from Research Diets (cat #D12108, New Brunswick, NJ). For glucose treatment, C57BL/6 mice were fasted 16 h, and 40% glucose (8 g/kg) was administered twice with 3 hours interval through oral gavage. Unless otherwise stated, male mice were used and all mice were fasted for 5–6 hours prior to euthanization using Isoflurane (Henry Schein, NY). All the animal studies have been approved by the Institutional Animal Care and Use Committee at Northeast Ohio Medical University.

### RNA isolation and quantitative real-time PCR

Total RNA was isolated using TRIzol Reagent (Life Technologies, NY). Reverse transcription and qPCR were performed as described previously [26]. Relative mRNA levels were calculated using the comparative cycle threshold (*Ct*) method with experimental values normalized to the values of 36B4 (ribosomal protein, large, P0; Rplp0) [27].

### Primary hepatocyte isolation

Mouse primary hepatocytes were isolated as described previously [28], [29]. In brief, mice were anaesthetized by intraperitoneal injection of 50 mg/kg pentobarbital. The portal vein was cannulated with a 23-gauge plastic cannula. Mouse livers were perfused with perfusion buffer I containing 10 mM HEPES, 0.15 M NaCl, 0.42 g/L KCl, 0.99 g/L glucose, 2.1 g/L NaHCO_3_, and 0.19 g/L EDTA. Simultaneously, the inferior vena cava was cut open. Subsequently, livers were perfused with collagenase buffer containing CaCl_2_ and collagenase (Sigma; St. Louis, MO). Primary hepatocytes were released from the Glisson capsule and collected in 50 mL centrifuge tubes. After serial centrifugations and washings, cells were cultured in 6-well plate pre-coated with collagen in 2 mL of DMEM supplemented with 10% FBS.

### Chromatin immunoprecipitation (ChIP) assay

200 mg liver tissues were used for ChIP assays as described previously [5], [30], [31] and following the manufacturer's instructions (cat# 17-295, Millipore, MA). Antibodies against acetyl-H3 (Lys9) and acetyl-H4 (Lys16) (Cell Signaling Technology, MA) were used to immuno-precipitate chromatins. Normal IgG was used as a measure of nonspecific background in immunoprecipitation. Chromatin purified from 10% sonicated tissue lysate was used as “input”. Real-time PCR was performed to test the chromatin enrichment in the CES1 promoter region. Chromatin enrichment was determined based on the fold change between critical threshold (C_T_) of specific antibody and C_T_ of normal IgG [ΔC_T_ =  C_T_(specific antibody)-C_T_(normal IgG)]. The derived ΔC_T_ was then normalized to C_T_ of the input. The primer sequences are AAACTCTAGGTCGGTGTGAC (forward) and TGCCCCACAGCTATAAACTC (reverse), which amplified a fragment between −148 bp and −14 bp in the *Ces1* gene promoter.

### Western blotting

Tissues were homogenized in ice-cold modified RIPA buffer and protein concentrations were determined using a Pierce BCA Protein Assay Kit (Thermo Scientific, IL). Western blotting was performed as described previously [30]. An antibody against mouse CES1 (Ces1g) was purchased from Abcam (Cambridge, MA). Antibodies against P-AKT, and AKT were purchased from Cell Signaling Technology.

### Recombinant adenovirus

Adenoviruses expressing ChREBP [32], Ces1 (Ces1g, Es-x) [5], shCes1 (Ces1g, Es-x) [5] and shAcl [33] have been described previously. Adenoviruses were grown in 293A cells, followed by subsequent purification by cesium chloride gradient centrifugation. About 1-2x10^9^ plaque formation units (pfu) of adenoviruses were injected into each mouse intravenously.

### Transient transfection assays

Transient transfections were performed in triplicate as described [34]. Briefly, pGL3-Ces1 luciferase reporter constructs were transfected into HepG2 cells, followed by treatment with either 5.5 mM glucose or 27.5 mM glucose. After 36 h, luciferase activities were determined and normalized to β-galactosidase activity.

### Plasma glucose analysis

Plasma glucose levels were measured using Infinity reagent from Thermo Scientific (Waltham, MA) or a glucometer (Onetouch).

### Statistical Method

The data were analyzed statistically using unpaired Student's *t*-test (two-tailed) and ANOVA (for more than two groups), followed by a post hoc Newman-Keuls test. The data were expressed as mean±SE. Only *p*<0.05 was considered statistically significant.

## Results

### Hepatic CES1 is regulated by nutritional status

**Figure 1:**
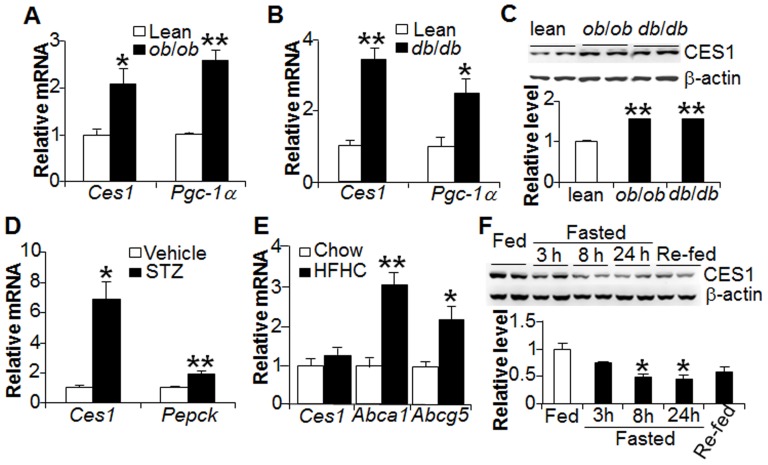
Hepatic CES1 is regulated by nutritional status. (A–C) Hepatic mRNA levels in *ob/ob* (A) and *db*/*db* mice (B) mice were determined by qRT-PCR and protein levels determined by Western blot assays (C, top) (n = 4–6 mice per group). In (C, bottom), protein levels were also quantified by Image J. (D) C57BL/6 mice were treated with either vehicle (0.1 M sodium citrate, pH 4.5) or streptozotocin (STZ) (50 mg/kg/d) for 5 days. Seven days after STZ treatment, mice were euthanized and hepatic mRNA levels were quantified (n = 5 mice per group). (E) Wild-type mice were fed a chow or high fat/high cholesterol (HFHC) diet (21% fat, 1.5% cholesterol) for 3 weeks and hepatic mRNA levels were determined (n = 8 mice per group). (F) C57BL/6 mice were fed a chow diet, or fasted for 3, 8, 24 h, or fasted for 24 h followed by refed for 24 h (n = 5 mice per group). Hepatic protein levels were determined by Western blot assays (top) and then quantified (bottom). Pgc-1α, peroxisome proliferator-activated receptor gamma coactivator-1α. Abca1, ATP-binding cassette (ABC) transporter A1. Abcg5, ABC transporter G5. Pepck, phosphoenolpyruvate carboxykinase. *p<0.05, **p<0.01.

To determine whether nutritional status affects CES1 expression, we first determined hepatic CES1 expression in diabetic mice. Our data indicated that hepatic *Ces1* mRNA (Figure 1A, B) and protein (Figure 1C) levels were significantly induced in both diabetic *ob*/*ob* mice and *db*/*db* mice. *ob*/*ob* mice and *db*/*db* mice are type 2 diabetes mouse models. Therefore, we also investigated the expression of CES1 in a type 1 diabetes mouse model. In streptozotocin (STZ)-treated mice, hepatic *Ces1* mRNA levels were induced by>7 fold (Figure 1D). Peroxisome proliferator-activated receptor gamma coactivator-1 α (PGC-1α) and phosphoenolpyruvate carboxykinase (PEPCK) served as positive controls. To investigate whether hepatic CES1 expression is affected by a Western diet, we fed C57BL/6 mice a high fat/high cholesterol (HFHC) diet for 3 weeks; the data show that treatment with an HFHC diet for a short time did not regulate hepatic *Ces1* expression (Figure 1E). ATP-binding cassette (ABC) transporter A1 (ABCA1) and ABC transporter G5 (ABCG5), which are known to be induced by dietary cholesterol [35], served as positive controls. Finally, we investigated the effect of fasting on hepatic CES1 expression. The data of Figure 1F show that fasting for 8 or 24 hours caused a reduction in hepatic CES1 protein levels. These data indicated that hepatic CES1 is regulated by nutritional status and glucose may induce hepatic CES1 expression.

### Hepatic CES1 is regulated by glucose but not insulin

**Figure 2:**
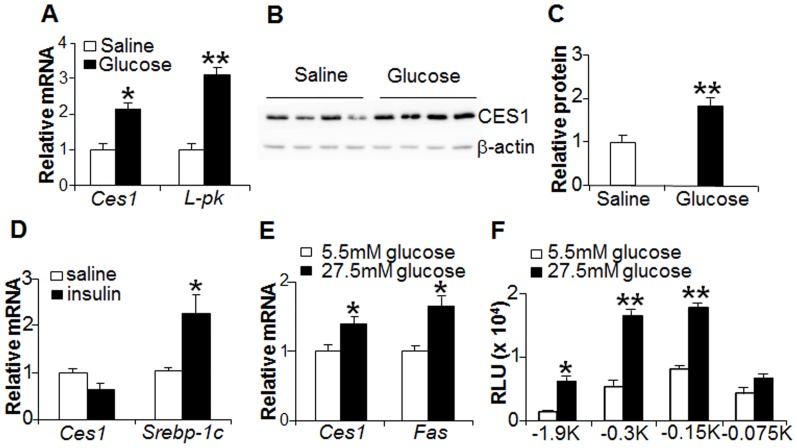
Hepatic CES1 is regulated by glucose but not insulin. (A–C) C57BL/6 mice were fasted for 16 h, followed by gavage with saline or glucose (8 g/kg) (n = 6 mice per group). Three hours after second oral gavage, hepatic mRNA levels (A) and protein levels (B) were determined. Hepatic CES1 protein levels were quantified (C). *L-PK* serves as a positive control in (A). (D) C57BL/6 mice were fasted for 16 h, followed by i.p. injection of either saline or insulin (0.8 units/kg) (n = 5 mice per group). After 3 h, mice were euthanized. *Srebp-1c* serves as a positive control. (E) Mouse primary hepatocytes were isolated and cultured in dulbecco's modified eagle medium (DMEM) plus 10% fetal bovine serum (FBS) overnight, followed by serum-free fasting for 8 h. Cells were then treated with either normal (5.5 mM) or high (27.5 mM) glucose for additional 24 h prior to quantification of mRNA levels. *Fas* serves as a positive control. (F) CES1 promoter-luciferase constructs were transfected into HepG2 cells, then treated with 5.5 mM or 27.5 mM glucose. After 36 h, luciferase activity was determined. Srebp-1c, sterol response element binding protein-1c. L-PK, liver type pyruvate kinase. Fas, fatty acid synthase. RLU, relative luciferase units. *p<0.05, **p<0.01.

To test our hypothesis that glucose induces hepatic CES1 expression, saline or glucose (8 g/kg) were administered to C57BL/6 mice twice with 3 hours interval via oral gavage. Mice were sacrificed 3 hours after second oral gavage and hepatic CES1 expression was determined. Hepatic *Ces1* mRNA levels (Figure 2A) and protein levels (Figure 2B, C) were induced by 2 fold in response to glucose stimulation. In contrast, insulin did not induce hepatic CES1 expression (Figure 2D).

In mouse primary hepatocytes, high glucose (27.5 mM) induced hepatic *Ces1* mRNA expression (Figure 2E), suggesting that glucose can directly regulate CES1 expression. To test whether glucose can directly regulate *Ces1* promoter activity, luciferase reporter assays were performed using a serial of luciferase reporter constructs with 5′-deletions. The data show that glucose stimulated *Ces1* promoter activity through a region between 75 bp and 150 bp upstream of the transcription start site (Figure 2F). Collectively, the data of Figure 2 demonstrate that glucose induces CES1 expression both *in vivo* and *in vitro*.

### ACL is required for glucose-induced hepatic CES1 expression

**Figure 3:**
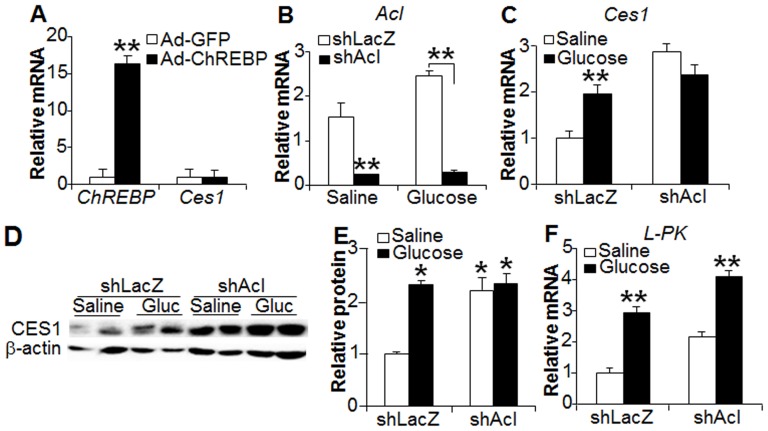
ACL is required for glucose-induced hepatic CES1 expression. (A) C57BL/6 mice were injected i.v. with adenovirus expressing GFP or ChREBP. After 5 days, hepatic mRNA levels were determined by qPCR (n = 7 mice per group). (B–F) C57BL/6 mice were injected with adenovirus expressing shLacZ or shAcl (n = 6 mice per group). After 5 days, mice were gavaged with either saline or glucose (8 g/kg). Hepatic mRNA levels of *Acl* (B), *Ces1* (C) and *L-pk* (F) were determined. Hepatic protein levels were determined by Western blot assays **(D)** and CES1 protein levels quantified (E). *p<0.05, **p<0.01.

ChREBP is suggested to be the principal mediator of glucose-induced gene expression in the liver [15], [16], [17]. Absence of ChREBP leads to the failure of glucose to induce hepatic glycolytic gene (*L-PK*) and lipogenic genes (*ACC* and *FAS*) [36]. However, over-expression of ChREBP in the liver had no effect on hepatic *Ces1* expression (Figure 3A), suggesting that glucose induces hepatic CES1 expression independent of ChREBP. It has been shown that ACL is required for glucose-mediated histone acetylation and gene activation [21], [22]. Thus, we investigated the role of ACL in glucose-mediated hepatic CES1 expression. Adenovirus-mediated expression of *Acl* shRNA reduced hepatic *Acl* mRNA levels by>85% (Fig. 3B). Interestingly, glucose induced hepatic CES1 mRNA and protein expression in the control mice but not in *Acl*-deficient mice (Figure 3C–E). *L-PK*, a gene responsive to glucose stimulation, served as a positive control (Figure 3F). Hence, the data of Figure 3 indicate that ACL is required for glucose to induce hepatic CES1 expression.

### ACL is required for glucose-mediated acetylation of histones (H3, H4) in the *Ces1* chromatin

**Figure 4:**
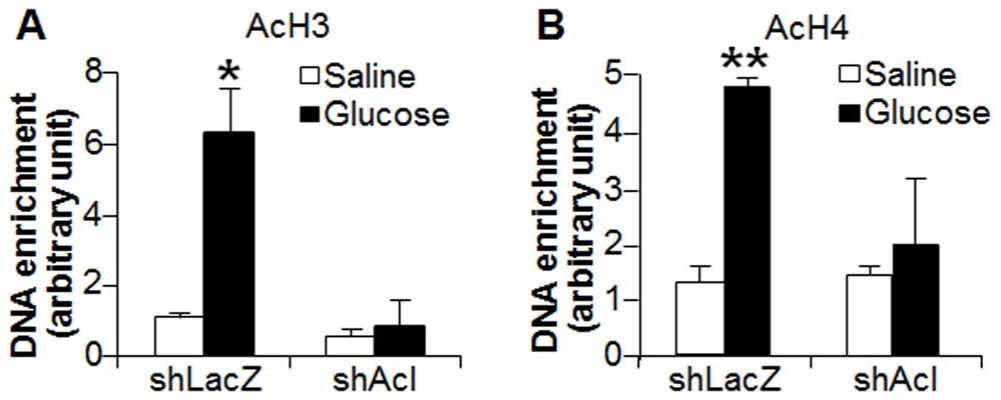
ACL is required for glucose-mediated acetylation of histones (H3, H4) in the CES1 chromatin. (A, B) C57BL/6 mice were treated as described in Fig. 3B–F. Liver lysates were used for ChIP assay to determine acetylation of histone 3 (AcH3) (A) and histone 4 (AcH4) (B). *p<0.05, **p<0.01.

Several lines of evidence have shown that glucose may regulate gene expression via epigenetic modifications [20], [21], [37], [38]. ACL converts glucose-derived citrate to acetyl-CoA, which subsequently serves as substrate for histone acetyltransferase for acetylation of H3 and H4 tails. Our data showed that glucose increased the acetylation of histone 3 and histone 4 in the *Ces1* chromatin and these effects were abolished in *Acl*-deficient mice (Figure 4A, B), indicating that ACL is required for glucose-mediated acetylation of histones (H3 and H4) in the *Ces1* chromatin.

### CES1 regulates postprandial glucose levels

**Figure 5:**
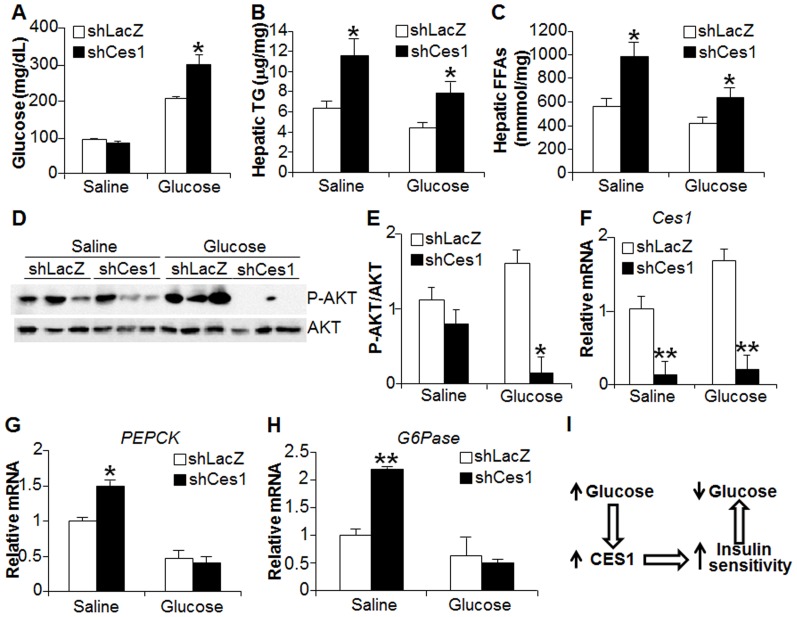
CES1 regulates postprandial levels. (A–H) C57BL/6 mice were injected with Ad-shLacZ or Ad-shCes1. After 5 days, mice were fasted for 16 h followed by gavage with saline or glucose (8 g/kg) (n = 6 mice per group). Blood glucose levels were measured 1 h after gavage using a glucometer (A). Mice were then sacrificed 3 hours after gavage. Hepatic triglyceride (TG) (B) and free fatty acid (FFA) (C) levels were analyzed. Hepatic protein levels were assessed by Western blot assays (D) and then the ratio of p-AKT to total AKT was quantified (E). Hepatic mRNA levels of *Ces1* (F), *PEPCK* (G) and *G6Pase* (H) were determined by qRT-PCR. (I) Reciprocal regulation between plasma glucose and hepatic CES1. Elevated plasma glucose induces hepatic CES1, which in turn helps lower plasma glucose levels likely via increasing peripheral insulin sensitivity. AKT, protein kinase B. *p<0.05 **p<0.01.

Postprandial blood glucose levels are tightly controlled to avoid any unwanted side effect of glucose. The finding that glucose induces CES1 expression and that CES1 regulates glucose metabolism [5] suggest that hepatic CES1 may regulate postprandial blood glucose levels. To test this hypothesis, C57BL/6 mice were injected with Ad-shLacZ and Ad-shCes1. Five days after adenovirus injection, mice were fasted for 16 h prior to gavage with saline or glucose (8 g/kg). Blood glucose levels were measured 1 h after gavage. For saline treatment, *Ces1*-deficient mice had similar blood glucose levels compared with the control mice (Figure 5A). For glucose treatment, however, *Ces1*-deficient mice had significantly higher blood glucose levels compared to the control mice (Figure 5A). In the liver, expression of *Ces1* shRNA resulted in elevated levels of hepatic triglycerides (TG) (Figure 5B) and free fatty acids (FFAs) (Figure 5C). Consistent with the latter data, hepatic *Ces1* knockdown reduced the ratio of phospho-AKT (p-AKT) to total AKT (Figure 5D and 5E). Finally, knockdown of hepatic *Ces1* reduced hepatic *Ces1* mRNA levels by>90% (Fig. 5F), and increased hepatic PEPCK and glucose 6-phosphatase (G6Pase) expression in saline- but not glucose-treated mice (Figure 5G and 5H). These latter data suggest that hepatic CES1 deficiency may cause hepatic insulin resistance and that the increase in postprandial glucose levels may not be a result of uncontrolled hepatic glucose production. Together, the data of Figure 5 demonstrate that hepatic CES1 plays an important role in regulating postprandial glucose levels.

## Discussion

CES1 is highly expressed in the liver. Our previous data show that hepatic CES1 regulates lipid and carbohydrate metabolism; increased hepatic CES1 expression reduces hepatic TG levels and lowers blood glucose levels [5]. CES1 has TG hydrolase activity. CES1 reduces hepatic TG levels via increasing TG hydrolysis and subsequent increase in fatty acid oxidation (FAO) [5]. In contrast, loss of hepatic CES1 increases hepatic TG levels through increasing lipogenesis [5]. Lipid homeostasis has a profound impact on insulin sensitivity and glucose metabolism. Plasma free fatty acid (FFA) levels correlate negatively to the degree of insulin sensitivity [39], [40]. Due to the regulatory role of CES1 in TG hydrolysis and FAO, it is not surprising to see that increased hepatic CES1 expression lowers plasma glucose levels and improves insulin sensitivity. In the present study, we investigated the regulation of hepatic CES1 by glucose and the physiological role of such regulation. Our data reveal a novel role for hepatic CES1 in postprandial glucose control.

Poor control of postprandial glucose is a significant contributor to type 2 diabetes mellitus. Persistent, moderate increase in postprandial glucose levels is a significant risk factor for macrovascular complications, and is more indicative of atherosclerosis than fasting glucose [23], [24]. In light of the risks of postprandial hyperglycemia for vascular events, tight control of postprandial glucose levels is important for long-term indices of diabetes control. After the start of a meal, blood glucose levels are increased. The increased blood glucose levels induce hepatic CES1 expression, which in turn helps lower blood glucose levels likely by increasing peripheral insulin sensitivity (Figure 5I). This conclusion is supported by the findings that increased hepatic CES1 expression lowers plasma glucose levels whereas loss of hepatic CES1 results in increased postprandial blood glucose levels.

We have previously shown that farnesoid X receptor regulates CES1 expression [5]. Our present study shows that hepatic CES1 is also regulated under physiological and pathological conditions. Under these latter conditions, hepatic CES1 expression is altered likely due to the change in plasma glucose levels. Indeed, our data show that glucose induces CES1 expression both *in vitro* and *in vivo*. Consistent with our finding, very recent data by Xiong *et al.* also show that glucose induces the expression of carboxylesterases in mouse primary hepatocytes [41].

Several lines of evidence suggest that glucose regulates gene expression through epigenetic modifications [20] and that nuclear ACL is important for glucose-mediated histone acetylation [21]. In this study, we show that glucose induces CES1 expression by epigenetic modifications of the acetylation status (H3 and H4) of the CES1 chromatin in an ACL-dependent manner. The histone tails interact with a region about 150 bp upstream of the transcription start site of CES1. Consistent with this finding, the data from the luciferase-promoter assays show that this region is required for glucose to induce CES1 promoter activity. Thus, glucose induces CES1 expression via acetylation of H3 and H4 in the CES1 chromatin.

Although our data show that hepatic CES1 is required for regulating postprandial glucose levels, the underlying mechanism remains to be fully determined. Global *Ces1* ^−/−^ mice have elevated plasma levels of triglycerides, free cholesterol, FFAs and insulin [6]. These mice also display insulin resistance, which results from reduced insulin sensitivity in both skeletal muscle and white adipose tissue [6]. However, CES1 is not expressed in skeletal muscle and its expression level in white adipose tissue is low (data not shown). Thus, insulin resistance observed in global *Ces1* ^−/−^ mice likely results from a deficiency in hepatic *Ces1*. Consistent with this speculation, our data show that hepatic *Ces1* deficiency results in impaired postprandial glucose clearance. Although hepatic CES1 deficiency may cause hepatic insulin resistance, skeletal muscle and white adipose tissues are the major organs responsible for plasma glucose clearance. Therefore, hepatic *Ces1* deficiency may affect peripheral insulin sensitivity. To precisely understand how hepatic CES1 deficiency regulates glucose homeostasis or insulin sensitivity, hyperinsulinemic-euglycemic clamp studies will be needed to help characterize the underlying mechanism.

In summary, the present study suggests a glucose-CES1-glucose cascade, in which glucose induces hepatic CES1 expression, which in turn lowers blood glucose levels. This cascade plays an important role in regulating postprandial glucose levels. Since high levels of postprandial blood glucose contribute to macrovascular complications, CES1 may be targeted for prevention of vascular diseases associated with hyperglycemia.
